# ELECT: prospective, randomized trial comparing microvascular plug versus platinum-fibered microcoils for embolization of aneurysm sac side branches before endovascular aortic aneurysm repair

**DOI:** 10.1186/s42155-024-00454-6

**Published:** 2024-05-03

**Authors:** Manuela Konert, Andrej Schmidt, Daniela Branzan, Tim Wittig, Dierk Scheinert, Sabine Steiner

**Affiliations:** 1 Division of Angiology, Department of Angiology, University Hospital Leipzig, Liebigstraße 18, 04103 Leipzig, Germany; 2 Department of Vascular Surgery, University Hospital Leipzig, Leipzig, Germany; 3 Helmholtz Institute for Metabolic, Obesity and Vascular Research (HI-MAG) of the Helmholtz Center Munich at the University of Leipzig and University Hospital Leipzig, Leipzig, Germany

**Keywords:** Embolization, Endoleak, Aortic aneurysm, Lumbar artery, EVAR, Plug, Coil

## Abstract

**Background:**

Preemptive selective embolization of aneurysm sac side branches (ASSBs) has been proposed to prevent type II endoleak after endovascular aortic aneurysm repair (EVAR). This study aimed to explore if an embolization strategy using microvascular plugs (MVP) reduces intervention time and radiation dose compared to platinum-fibered microcoils. Furthermore, the effectiveness of the devices in occluding the treated artery was assessed.

**Methods:**

Sixty patients scheduled for EVAR underwent percutaneous preemptive embolization of ASSBs using MVPs or coils after a 1:1 randomization. Follow-up imaging was performed during aortic stentgraft implantation.

**Results:**

Overall, 170 ASSBs were successfully occluded (83 arteries by MVPs and 87 by coils) and no acute treatment failure occurred. The mean procedure time was significantly lower in the group treated with MVPs (55 ± 4 min) compared to coil occlusion (67 ± 3 min; *p* = 0.018), which was paralleled by a numerically lower radiation dose (119 Gy/cm^2^ vs. 140 Gy/cm^2^; *p* = 0.45). No difference was found for contrast agent use (34 ml MVP group vs 35 ml coil group; *p* = 0.87). At follow-up, reopening of lumbar arteries was seen in nine cases (four after coil embolization; five after MVPs).

**Conclusion:**

Both microvascular plugs and coils can be effectively used for preemptive embolization of aneurysm sac side branches before EVAR. Use of plugs offers a benefit in terms of intervention time.

**Trial registration:**

ClinicalTrials.gov Identifier: NCT03842930 Registered 15 February 2019.

**Graphical Abstract:**

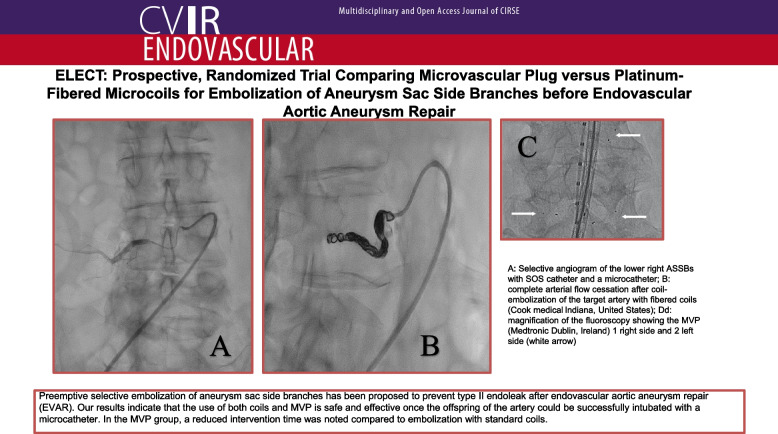

## Introduction

As an alternative to open repair of abdominal aortic aneurysm (AAA), the endovascular aortic aneurysm repair (EVAR) by stentgraft implantation has emerged as a less invasive alternative over the last decades. While EVAR has been found to be associated with a lower 30-day mortality and morbidity compared to open surgery [1], the development of endoleaks with a persistent perfusion of the aneurysm sac requiring re-interventions, has been identified as a major limitation of the technique [2, 3]. Type I endoleaks (leak at graft ends, causing an inadequate seal) and type III endoleaks (leak through a defect in the graft fabric) are associated with adverse clinical outcomes and are therefore considered treatment failures with an immediate need for repair. In contrast, the clinical relevance of type II endoleaks defined as sac filling via lumbar arteries or the inferior mesenteric artery, which have been reported to occur in up to 20–30% of patients during follow-up, remains controversial [4–6]. Recently, long-term results of the largest randomized trial, EVAR-1, comparing open repair to EVAR has shown an increased rate of late rupture for EVAR [1] and it cannot be ruled out, that type II endoleaks may play a causative role for this process [7, 8]. Importantly, in clinical routine, type II endoleaks are often identified in case of late sack enlargement. Subsequent endovascular treatment of type II endoleaks is complex with a relatively low reported success rate of about 63% with a high variation (15–89%) [9]. An alternative strategy would be to embolize all relevant aneurysm sac side branches (ASSBs/lumbar arteries and inferior mesenteric artery), that could potentially lead to type II endoleaks, before EVAR [10]. So far, no clear consensus exists on different strategies for the prevention and treatment of type II endoleaks [11]. Some centers advocate the use of prophylactic visceral artery and lumbar artery (LA) embolization, whereas others intervene only in case of aneurysm enlargement post EVAR during follow-up [11, 12].

For preemptive embolization, coils have been typically used as standard devices. Coils are small platinum spirals that—when released into the vessel—create a thrombogenic environment due to slowed flow, ultimately leading to vessel occlusion. There are limited data on the efficacy of standard coils to achieve complete occlusion of the treated artery in the long term. Some reports showed late reopening of arteries after coil embolization, for example after treatment of gastrointestinal bleeding [13, 14].

As an alternative, smaller plugs have been developed for use in vessels with small diameter. The microvascular plug (MVP, Medtronic, Dublin Ireland) is a new embolization device used to occlude arteries of small and middle caliber (1.5 up to 5 mm in diameter). It is a cage made of nitinol, whose proximal segment is covered with a polytetrafluoroethylene (PTFE) membrane. Embolization targets reported in the literature include hypogastric and aortoiliac aneurysms, pulmonary and renal arteriovenous malformations and acute hemorrhage [15]. In comparison to coils, MVPs are easier to use, mainly because only one device per artery is needed instead of several standard coils. Thus, a reduction of fluoroscopy time and procedure time as well contrast medium usage can be expected using MVPs compared to standard coils.

The use of the MVP for ASSBs embolization in order to prevent type II endoleaks after EVAR has not been described, yet. This study aimed to explore if an embolization strategy using MVP reduces intervention time and radiation dose compared to platinum-fibered microcoils. Further, the effectiveness of the devices in occluding the treated artery was determined.

## Methods

### Study design and patient population

The ELECT study is a prospective, single-center, 1:1 randomized trial to compare the radiation dose measured as a dose-area product [DAP] as well as intervention time with the MVP microvascular plug (Medtronic, Dublin Ireland) versus platinum-fibred coils (MicroNester Embolization, Cook Medical, Indiana, United States) for embolization of ASSBs before endovascular aortic repair.

All patients between April 2019 and June 2021 with an infrarenal or juxtarenal aortic aneurysm and an indication for an endovascular aortic repair were eligible, if a prior CT scan had identified at least two patent lumbar arteries with a minimum diameter of 2 mm in the area of the aneurysm. Patients were routinely scheduled for embolization before EVAR according to the local standard of care. Exclusion criteria comprised patients with any other aortic pathology, major untreated cardio-pulmonary disease, or a life-expectancy of less than one year as well as patients with a severe contrast agent allergy, severe reduction in glomerular filtration rate (chronic kidney disease stage 4 or higher) and impaired thyroid function, if not under stable treatment.

Additionally, we used stratification for body mass index (BMI) to address another major factor in radiation dose: obese patients. In order to gain a stable and clear visualization, modern angiographic systems automatically elevate the voltage. This results in a higher radiation dose, especially in obese patients in the abdominal area.

Ethics approval was obtained from the University of Leipzig Ethical Committee (331/18-ek) and patients provided written informed consent before enrolment.

Patient randomization was conducted using dedicated software at an outsourced independent data coordinating center (www.randomizer.at). Participants were randomly allocated in 2 groups (coil vs. MVP) using a stratified randomization procedure with matched subjects in each group based on BMI (BMI ≤ 30 and overweight, BMI > 30 obese). Due to the permuted block size of 4, 31 patients were randomized in the MVP group and 29 in the coil group.

### Embolization procedure

**Fig. 1:**
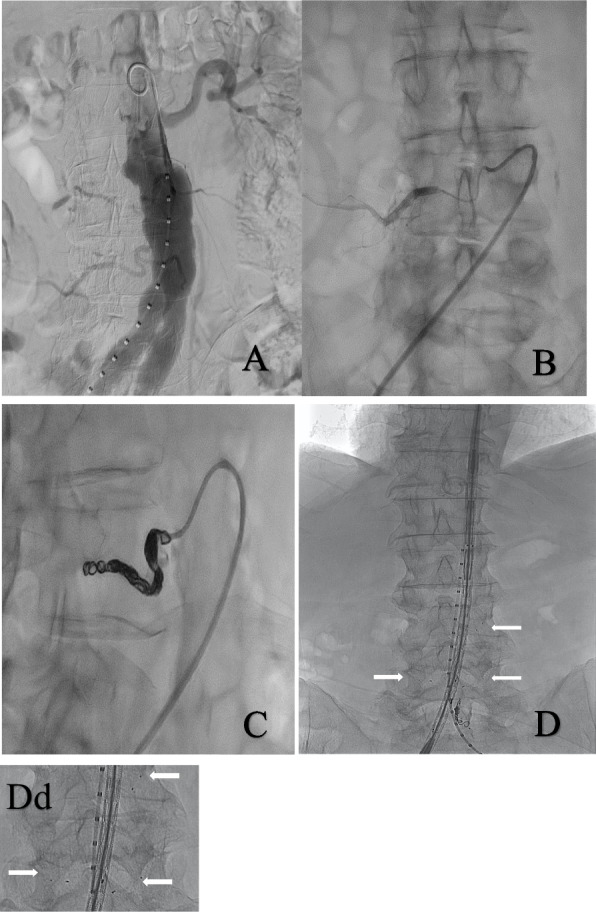
Patient 1 (**A**-**C**) Embolization with coils, **A** Angiogram of the Aorta with a pigtail catheter showing 4 patent ASSBs (L3 and L4 both sides) and the left renal artery, the IMA and the left renal artery; **B** Selective angiogram of the lower right ASSBs with SOS catheter and a microcatheter; **C** Complete arterial flow cessation after coil-embolization of the target artery with fibered coils (Cook medical, Indiana, United States); Patient 2 (**D** and **Dd**) Embolization with MVP, **D** Stentgraft inserted via the right groin and the pigtail-catheter to perform an angiography via left groin. MVP (Medtronic Dublin, Ireland) (white arrow) in the lumbar arteries L4 right and L4 and L3 left side, occlusion of the inferior mesenteric artery with coils, **Dd** Magnification of the same picture

Arterial access with a 6-French-sheath (Radiofocus Introducer; Terumo, Tokyo, Japan) was obtained via the groin, puncturing the common femoral artery under ultrasound guidance. Typically, in telescope technique with a 6-French LIMA-guiding catheter (Boston Scientific, Marlborough, Massachusetts, US) and a 5-French SOS-catheter (AngioDynamics, Latham, New York, US) the orifice of the lumbar arteries in the aneurysmatic region of the aorta was localized, which was identified in advance by a CT scan. The enrolled subjects were randomly assigned either to MVP- microvascular plug (study group) or to the platinum-fibred coils (control group), once the orifice of the first target artery was intubated. After randomization, measurement of the intervention time and the radiation dose for the occlusion of the lumbar artery started via a microcatheter (Progreat 2.9Fr, Terumo, Tokyo, Japan), introduced into the SOS-catheter. In case of randomization to the MVP arm, one device per artery was used. We used the MVP-3 for vessels sized 1,5-3 mm in diameter and the MVP-5 for vessels sized 3-5 mm in diameter. In the coil arm, the number and size of coils used was at the discretion of the operator, with the goal to occlude the target vessel. The intervention was performed by three operators with advanced experience in endovascular treatment of aortic aneurysm and embolization procedures. Once the vessel was occluded, defined by the reduced flow of the contrast agent in fluoroscopic control, the time and radiation dose registration were stopped until the next lumbar artery was successfully accessed. In case a second session was deemed necessary, at least 4–6 weeks were scheduled between sessions to allow sufficient collateral network development. Figure 1 depicts the process of plug and coil embolization.

Embolization of the inferior mesenteric artery (IMA) was not included in our protocol due to the complex anatomy and the preferred use of vascular plugs for embolization.

After the embolization procedure, common femoral access closure and hemostasis were secured using either FemoSeal (Terumo, Tokyo, Japan) or Proglide (Abbott, Illinois, United States) at the operators’ discretion.

### Post-interventional care and medication

All patients received a duplex-ultrasound examination of the access-sites to rule out access-complications on the first post-procedural day. Antiplatelet therapy consisting of either aspirin (100 mg daily) or clopidogrel (75 mg daily) as well as statin therapy were prescribed in all patients for secondary prevention of cardiovascular disease.

### EVAR procedure

The final exclusion of the aneurysm sac with implantation of the aortic stentgraft was either performed during the same treatment-session, or as a second procedure days to weeks later. The choice of the stentgraft was left at the discretion of the operator. The procedure was performed via percutaneous femoral access.

### Study endpoints and clinical outcomes

Primary endpoint was the radiation dose (DAP in Gy/cm^2^) during the embolization of the lumbar arteries comparing a new embolization technique with MVP versus standard embolizations with coils. Key secondary endpoint was the intervention time for embolization of the lumbar arteries with the MVP versus coils.

During the EVAR-procedure, an angiography was performed routinely before stent graft-implantation to assist positioning of the graft. Based on this angiography, it was determined if the treated lumbar arteries were occluded, or re-perfusion had occurred.

### Statistical analysis

As this was a pilot study, we did not perform a formal sample size calculation. Sixty patients were aimed to be included, as this would support study inclusion and completion within one year.

Continuous data were presented as mean ± standard deviation, categorical data as numbers (percentage). Continuous variables were compared using appropriate tests, such as the unpaired Student’s t-test. Categorical variables were assessed using appropriate contingency table analyses (chi-square or Fisher’s exact test). Statistical analysis was performed using STATA (release 15, StataCorp LLC, College Station, Texas, US).

## Results

### Patient and procedural characteristics

We included 60 patients in this study with a mean age of 71 ± 1.1 years. The majority of them were male (52; 86,7%) with typical cardiovascular risk factors. A total of 51 patients were treated for arterial hypertension and 41patients for hyperlipidemia, 15 patients had a history of coronary artery disease. Detailed patient characteristics are summarized in Table 1.

**Table 1:** Demographics and clinical characteristics of the 60 patients in the study

	**MVP group** ***N* = 31 (%)**	**Coil group** ***N* = 29 (%)**
Sex		
Male	25 (41.7)	27(45)
Female	6 (10)	2 (3.3)
Age (years)	72 ± 1.6	70 ± 1.6
BMI > 30	16 (26.7)	14 (23.3)
Arterial hypertension	28 (46.7)	23 (38.3)
Diabetes mellitus	8 (13.3)	9 (15)
Hyperlipidemia	20 (33.3)	21(35)
CAD	4 (6.7)	11 (18.3)
PAD	5 (8.3)	1 (1.7)
Smoker	7 (11.7)	7 (11.7)
Aneurysm diameter (mm)	52 ± 2.4	53 ± 4.3

Data are given as number (percentage) or mean ± standard deviation

*MVP* microvascular plug, *BMI* Body Mass Index, *CAD* coronary artery disease, *PAD* peripheral artery disease, *mm* millimeter

The designated study device was successfully used in all assigned patients. For three target arteries, the ostium could not be intubated and therefore no embolization could be performed. Thus, these vessels were not included in the trial. Overall, we treated 170 lumbar arteries in 60 patients. In the coil group 87 ASSBs were treated and 83 ASSBs were treated with MVP.

As reported in Table 2 the ASSBs had an average size of 4 ± 0.14 mm in the MVP group and 4.3 ± 0.12 mm in patients treated with coils showing no significant difference between both groups. We used an average of 12.6 ± 8.6 coils to treat all planned vessels and an average of 2.8 ± 1.2 MVPs in the second group. The average size of the used coils was 4.8 ± 2.2 mm ranging from 3 to 8 mm. In the MVP group, we used 28 times the 5 mm plug and 6 times the 3 mm plug. In 16 patients, a staged approach with 2 coiling sessions was performed as we planned either to treat more than 4 vessels or had difficulties intubating the orifice during the first session.

**Table 2:** ASSBs embolization procedure characteristics

	**MVP group** *N* = 31(%)	**Coil group** *N* = 29(%)	*P*-value
Average size ASSBs (mm)	4.0 ± 0.14	4.3 ± 0.12	0.63
Average amount of devices used	12.6 ± 8.6	2.8 ± 1.2	< 0.005
Average size of devices (mm)	4.8 ± 2.2	4.6 ± 0.8	0.62
Embolization in single approach	26 (84)	19 (66)	-
Embolization in staged approach	5 (16)	10 (34)	-
Occlusion of IMA	8 (26)	7 (24)	-
Occlusion of MSA	3 (10)	2 (7)	-
Complications	0 (0)	0 (0)	-

Data are givens as mean ± standard deviation (SD) or number (percentage)

*MVP* microvascular plug, *ASSBs* aneurysm sac side branches, *mm* milimeter, *IMA* inferior mesenteric artery, *MSA* median sacral artery

In addition, we occluded the inferior mesenteric artery in 15 patients and the median sacral artery in five patients in the same session as shown in Table 2. There were no documented complications, such as access side complications, perforations, bleeding or dislocation of the embolization material.

### Radiation dose and intervention time

Mean procedure time measured from intubating the ostium of the artery until deploying the last embolization device was 55 ± 4 min in total using the MVP and 67 ± 3 min (*p* = 0.018), in total using the coils (Table 3).

**Table 3:** Procedural characteristics and results

	**MVP group** *N* = 31	**Coil group** *N* = 29	*P*-value
Procedure time (min)	55 ± 4	67 ± 3	0.018
Amount of contrast agent (ml)	34 ± 6	35 ± 6	0.87
DAP (Gy/cm^2^)	119 ± 17	140 ± 38	0.45

Data are givens as mean ± standard deviation (SD)

*MVP* microvascular plug, *DAP* dose area product, *Gy* Gray

As embolization is performed under fluoroscopic control and contrast media is only injected at the beginning and end of vessel embolization, no difference was found for contrast agent use (34 ml plug group vs 35 ml coil group; *p* = 0.87), which was paralleled by a numerically lower radiation dose (119 Gy/cm^2^ vs. 140 Gy/cm^2^; *p* = 0.45).

No adverse events occurred in this patient population until hospital discharge.

### Follow-up results and EVAR characteristics

From the total 170 lumbar arteries embolized, nine arteries (four after coiling, five after MVP embolization) exhibited re-established flow at the baseline angiogram during EVAR.

A total of 57 patients were treated with standard infrarenal stent-graft. We used Endurant (Medtronic, Dublin, Ireland) in 19 patients, Zenith alpha (Cook medical, Indiana, United States) in 14 patients, C3 (Gore, Delaware, US) in 13 patients, Ovation (Endologix, California, US) in 9 patients, 1 Altura (Lombard medical, United Kingdom) and 1 Anaconda (Terumo, Tokyo, Japan).

Three patients received fenestrated, custom-made stent-grafts, two from Cook medical (Indiana, US) and one from Artivion (CryoLife, Georgia, US).

No access side or stent-graft related complications were seen in the whole cohort. Stent-graft implantation was successful in all cases.

All patients are under surveillance in our outpatient clinic after aortic interventions and we perform CT scans on a regular base. Only in the case of sac expansion we would reintervene the patient.

## Discussion

Type II endoleaks are a major limiting factor for the long-term benefit and success after endovascular repair of abdominal aortic aneurysm. Type II endoleaks may not be as benign as considered. Seike et al. showed a correlation between persistent typ II endoleaks and late adverse events, including aneurysm sac enlargement, reintervention, rupture, and abdominal aortic aneurysm–related mortality after endovascular aneurysm repair [8].

Furthermore, type II endoleaks are associated with the absence of sac shrinkage. Not only an increase in aneurysm diameter after EVAR, but also the absence of sac shrinkage is associated with increased mortality compared to sac shrinkage [7]. Lopez et al. did show in the VASCUNExplanT Project that patient with endoleaks are the main reason for a conversion to open surgery after failed endovascular aortic aneurysm repair and patients with type II endoleaks represent the highest proportion [16].

We were recently able to show that preemptive coiling can achieve a remarkably high rate of sac shrinkage compared to the literature [10]. However, long-term results must first show whether the preemptive coiling strategy is beneficial for aneurysm patients. The technical feasibility, safety of the treatment and the additional radiation exposure will certainly influence a potential future change in the treatment strategy of AAA patients. However, there are no recommendations and little expertise on how embolization should be performed prior to EVAR.

So far, coils had been mainly used for embolization of ASSBs. One major concern regarding coil embolization is treated vessel recanalization, which compromises the durability of the treatment [14, 17]. As an alternative with potential better occlusion efficacy, plugs have been developed for clinical routine use.

Two prospective randomized studies compared the use of coils and vascular plug (Amplatzer Vascular Plug) as an embolic device. Guirola et al. compared the Amplatzer Plug (Abbott, Illinois, United States) versus coils for treatment of pelvic congestion syndrome [14]. The study showed a significantly longer radiation time (33.4 min. ± 4.68 vs 19.5 min. ± 6.14) and also a significantly higher dose of radiation (air Kerma 948.0 mGy ± 248.45 vs 320.7 mGy ± 134.33) for the coil-arm [17] Bulla et al. also compared the Amplatzer plug versus coils for the occlusion of the gastroduodenal artery before lodin therapy [18]. The authors were also able to show a significantly higher radiation time (23.1 min vs. 8.8 min) for coiling of the gastroduodenal artery compared to the use of the Amplatzer plug [18]. In this retrospective comparison, they could also show a higher effectiveness in the closure of the gastroduodenal artery by the vascular plug (3% vs. 26.9%) [18]. Due to its morphology, the MVP appears to be better suited for use in the much smaller lumbar arteries than the somewhat bulky Amplatzer plug.

So far, no prior studies focused on a direct comparison of MVP versus coils, especially not in the setting of preemptive ASSBs occlusion aiming to prevent type II endoleaks. In line with the aforementioned studies in different embolization setting, we also showed that using a plug is associated with a significantly shorter intervention time and less radiation exposure compared to standard coil use. The amount of contrast agent used where similar in both groups as the embolization process itself does not require additional contrast-guided imaging steps after target vessel access. In contrast to the study by Bulla and co-workers, our findings do not suggest a difference between the strategies for reopening rates, which were rare in both groups.

In our opinion, MVPs are a good alternative to coils for the embolization of ASSBs. They are safe to use and lead to shorter intervention times with less radiation exposure.

### Study limitations

The study was performed in a single center with limited follow-up to 4-6 weeks in average. As we know from different studies endoleaks may develop over time and even typ II endoleaks can cause a progress of the aortic diameter after EVAR. A longer follow-up in patients after embolization of the ASSBs will add valuable information to these limitations of endovascular treatment in patients with aortic aneurysm. All our patients are under surveillance in our outpatient clinic. We perform reinterventions in the case of type I or III endoleaks or in the case of sac enlargement for type II endoleaks. But the long-term follow up is not included in this study protocol. Another limitation is the type of coils we used. We used fibred platinum coils. Meanwhile, there are also large-volume coils (Penumbra, Alameda, California, US) and hydrogel coils (Azur, Terumo, Tokyo, Japan), which could have an influence on the intervention time and the reopening rate compared to fibred platinum coil. The number and size of coils used are to the discretion of the operator which can be seen as a bias. Our analyses shows that we treated 4 more ASSB´s with coils compared to MVP`s.

Regarding the cost effectiveness; MVP´s are more expensive than coils, but usually only 1 MVP per artery is used, whereas you need 4–5 coils per artery. The prices do depend according to the contract agreed by each hospital, as well as it differs between different countries. Therefore, it was decided that we cannot give an overall estimate and thus this data was removed from the study.

## Conclusion

Our results indicate that the use of both coils and MVP is safe and effective once the artery´s offspring could be successfully intubated with a microcatheter. In the MVP group, a reduced intervention time was noted compared to embolization with standard coils.

## Data Availability

The datasets used and analysed during the current study are available from the corresponding author on request.
